# Sporadic spinal psammomatous malignant melanotic nerve sheath tumor: A case report and literature review

**DOI:** 10.3389/fonc.2023.1100532

**Published:** 2023-02-24

**Authors:** Giulio Bonomo, Alessandro Gans, Elio Mazzapicchi, Emanuele Rubiu, Paolo Alimonti, Marica Eoli, Rosina Paterra, Bianca Pollo, Guglielmo Iess, Francesco Restelli, Jacopo Falco, Francesco Acerbi, Marco Paolo Schiariti, Paolo Ferroli, Morgan Broggi

**Affiliations:** ^1^ Department of Neurosurgery, Fondazione IRCCS Istituto Neurologico C. Besta, Milan, Italy; ^2^ School of Medicine, University of Milan, Milan, Italy; ^3^ Department of Neurological Surgery, Policlinico “G. Rodolico-S. Marco” University Hospital, Catania, Italy; ^4^ Molecular Neuro-Oncology Unit, Fondazione IRCCS Istituto Neurologico C. Besta, Milan, Italy; ^5^ Neuropathology Unit, Fondazione IRCCS Istituto Neurologico C. Besta, Milan, Italy

**Keywords:** malignant melanotic nerve sheath tumor, spinal, nerve sheath, tumor, psammomatous, sporadic, case report, melanotic schwannoma

## Abstract

**Background:**

Sporadic Spinal Psammomatous Malignant Melanotic Nerve Sheath Tumor (SSP-MMNST) is a rare subgroup of peripheral nerve sheath tumors arising along the spine. Only a few reports of SSP-MMNST have been described. In this paper, we review the literature on SSP-MMNST focusing on clinical, and diagnostic features, as well as investigating possible pathogenetic mechanisms to better implement therapeutic strategies. We also report an illustrative case of a young female presenting with cervicobrachial pain due to two SSP-MMNSTs arising from C5-6 right spinal roots.

**Case description:**

We report a case of a 28-year-old woman presenting with right arm weakness and dysesthesia. Clinical examination and neuroimaging were performed, and, following surgical removal of both lesions, a histological diagnosis of SSP-MMNST was obtained.

**Results:**

The literature review identified 21 eligible studies assessing 23 patients with SSP-MMNST, with a mean onset age of 41 years and a slight male gender preference. The lumbar district was the most involved spinal segment. Gross-total resection (GTR) was the treatment of choice in all amenable cases, followed in selected cases with residual tumor by adjuvant radiotherapy or chemotherapy. The metastatic and recurrence rates were 31.58% and 36.8%, respectively.

**Conclusion:**

Differently from common schwannomas, MMNST represents a rare disease with known recurrence and metastatization propensity. As reported in our review, SSP-MMNST has a greater recurrence rate when compared to other forms of spinal MMNST, raising questions about the greater aggressiveness of the former. We also found that residual disease is related to a higher risk of systemic disease spreading. This metastatic potential, usually associated with primary lumbar localization, is characterized by a slight male prevalence. Indeed, whenever GTR is unachievable, considering the higher recurrence rate, adjuvant radiation therapy should be taken into consideration.

## Introduction

Malignant melanotic nerve sheath tumor (MMNST) represents a rare variant of nerve sheath neoplasms and accounts for less than 1% of all primary peripheral nerve sheath tumors (1). MMNST predominantly develops from spinal or visceral autonomic nerves (2). Considering its histological and ultrastructural traits of schwannomian differentiation, MMNST was historically considered an atypical variant of schwannoma, hence the definition of “Melanotic schwannoma” (3). However, given its recently uncovered different genetic profile, MMNST now represents a distinct malignant entity, as reported in the 2020 World Health Organization (WHO) classification of soft tissue tumors and in the 2021 WHO classification of central nervous system (CNS) tumors (4, 5). Psammomatous MMNST is an even rarer variant of MMNST, often arising along with the gastrointestinal tract-related nerves or in the extremities (6). The first reported case of MMNST was that by Millar et al. in 1932. The Authors described an uncommon pigmented neural tumor stemming from a sympathetic nerve in the thoracic region (7). The coining of the term would come later, with the 1975 Fu et al. publication (8).

MMNSTs are categorized according to two main groups. The first one is represented by its association with Carney Complex (CC). This entity features autosomal dominant inheritance and is characterized by patchy skin pigmentation, endocrine hyperactivity, and cardiac, mammary, and cutaneous myxomas (9, 10). About 50% of all MMNSTs are associated with CC (9). The second group of MMNSTs includes the presence of round concentric calcifications, known as psammoma bodies. Around 50% of all MMNSTs are psammomatous, and about half of these cases are CC-related (11). Therefore, it is crucial to rule out CC in MMNST patients and consider the possibility of sporadic cases of the disease. Additionally, few reported cases were associated with neurofibromatosis (12).

MMNST displays both malignant and metastatic potential depending on its histology. Microscopically, MMNST features spindle-shaped and epithelioid cells in intertwining fascicles, with a significant concentration of melanin both in the neoplastic cells and in the associated melanophages (1).

Clinical presentation depends on anatomical location. MMNST typically arises along the spinal nerve sheath (13), thus explaining frequent symptoms such as pain and paraesthesia (14–17).

To date, the literature has reported approximately 150 cases of MMNST. In this paper, we review the literature on SSP-MMNST in order to improve the knowledge of this disease, focusing on clinical, and diagnostic data, as well as investigating possible pathogenetic mechanisms to better implement therapeutic strategies. We also report a case of a young female presenting with cervicobrachial pain due to two SSP-MMNSTs arising from C5 and C6 right roots.

## Results

**Figure 1:**
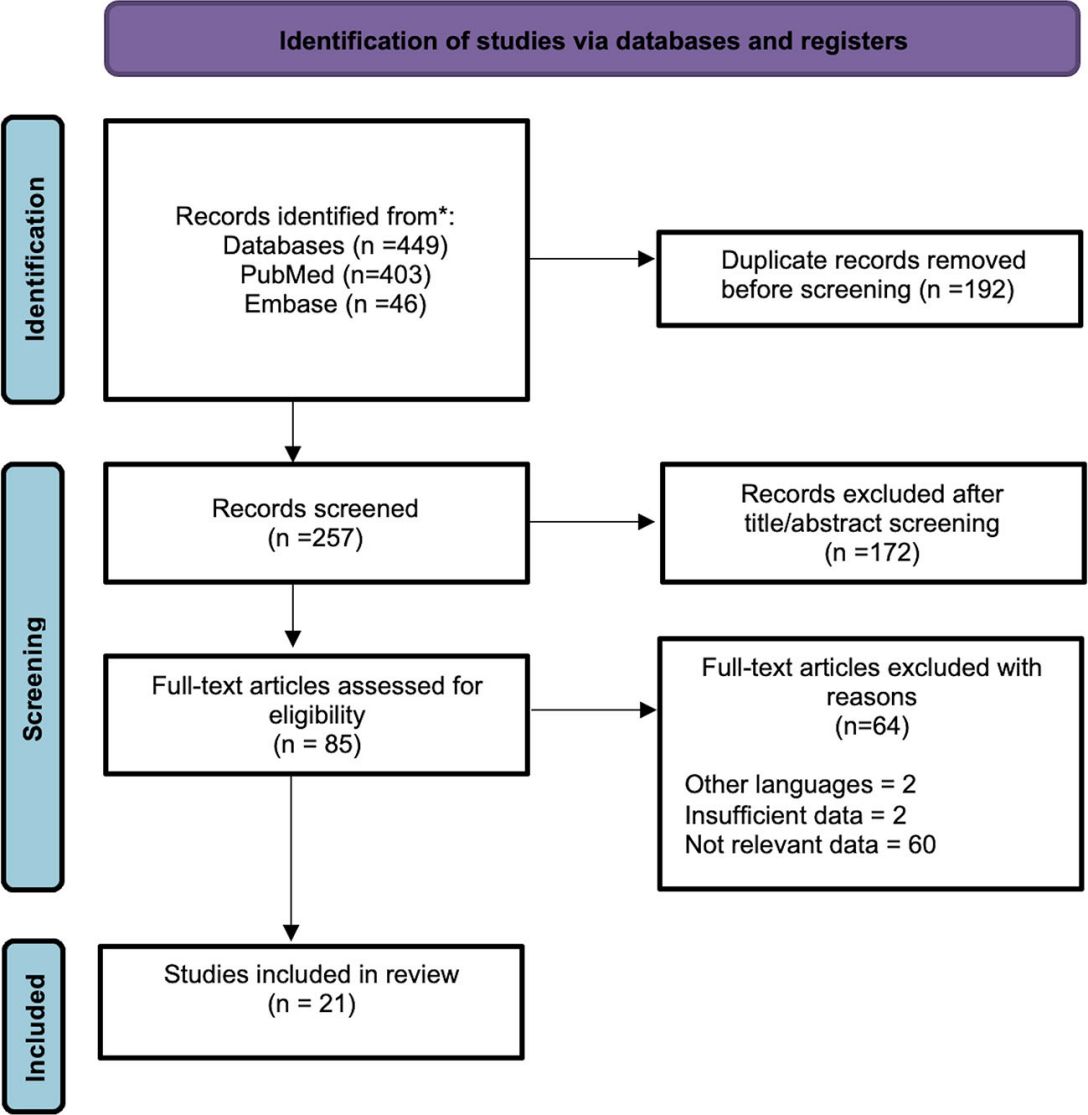
PRISMA flowchart diagram.

A PRISMA flowchart is presented in **Figure 1**. A primary search returned 449 records. After excluding 192 duplicates, 257 records were screened. After screening titles and abstracts and removing articles without full-text, 85 full-text articles were left and evaluated for eligibility. Given that 64 of them were ineligible for inclusion, we selected 21 full-text papers for the review. We identified 23 cases of SSP-MMNST, including 9 (39.13%) females and 14 (60.87%) males. The male-to-female ratio was 1.56:1, suggesting a slight male prevalence of the disease. The mean age of onset of the disease was 40.39 ± 13.11 years. Spinal localization was as follows: 4 cervical (16.67%), 7 thoracic (29.17%), 9 lumbar (37.5%), and 4 sacral (16.67%). Metastases were reported in 6 cases (4 male, 2 female) with a metastatic rate of 31.58%. Lungs were the main site of metastatization (50%), followed by spinal cord, meninges, bone, chest wall, lymph nodes, and brain parenchyma. In 83.33% of the metastatic cases, the primary tumor site was in the lumbar region, particularly in L4/L5. The mean age of metastatic patients was 37.33 ± 9.09 years. There was no significant difference between the mean age of metastatic and non-metastatic patients (p=0.519). Nonetheless, males appeared slightly more at risk of developing metastasis, with a relative risk (RR) of 1.45 (p=0.6082). Treatment approaches in patients included gross-total resection (GTR) in 17 cases (73.91%), and subtotal resection in 5 cases (22.73%), while one refused the treatment. Patients underwent adjuvant radiotherapy in 4 cases (18.18%), with only one without any recurrence. The disease recurrence rate among the 19 patients with known data was 36.84% (7/19). Of these 7 patients, 4 succumbed to the disease (57.14%), 2 were alive with disease stability (28.57%), and 1 was not reported. Of these 7 patients with disease recurrence, 4 had metastatic locations (57.14%). Finally, 3 of 5 (60%) patients who underwent subtotal resection (STR) developed disease recurrence. On the other hand, only 4 of the 17 patients that underwent GTR suffered a recurrence (23.53%). Considering all 23 patients with SSP-MMNST, the outcome was unknown in 6 patients, 9 were disease-free (52.94%), 6 died, and 2 were still affected by the disease at the time of follow-up. Follow-up was different for each of the cases.

## Case presentation

**Figure 2:**
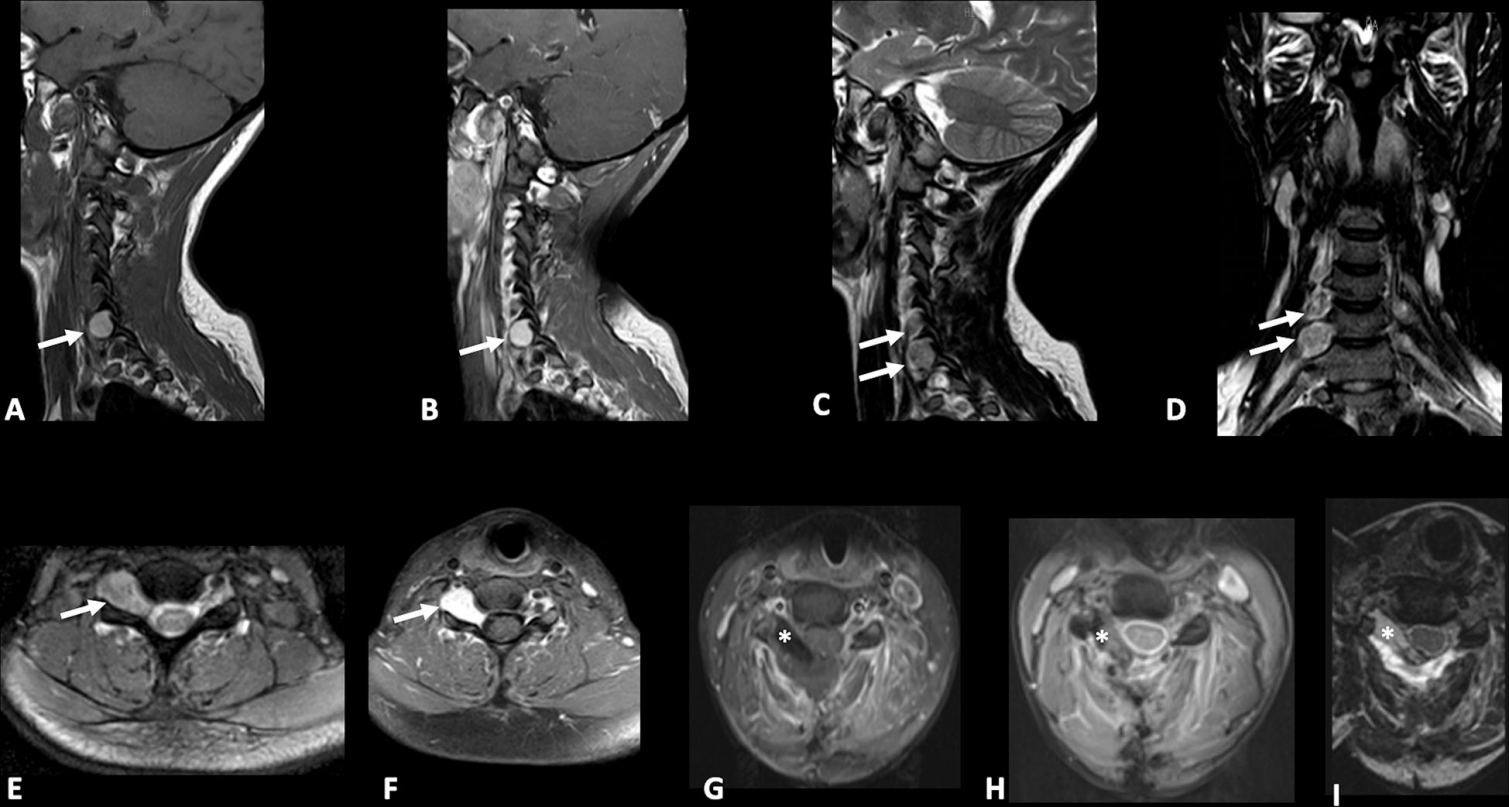
Pre-operative sagittal T1-weighted **(A)**, sagittal **(B)** and axial **(F)** post-gadolinium T1-weighted, sagittal **(C)**, coronal **(D)**, axial **(E)** T2-weighted Nuclear Magnetic Resonance (MRI) images demonstrating the presence of two right sided extradural spinal lesions (arrows) at C5-C7 levels one of which is larger with intraforaminal and paravertebral extension and “dumbbell” shape. Postoperative axial post-gadolinium T1-weighted and fat suppression **(G)**, Gradient echo (GRE) **(H)**, and T2-weighted **(I)** MRI images demonstrating gross total resection (GTR) of lesions and placement of fat tissue (asterisk) to reinforce dural closure.

A 28-year-old woman presented with a one-year history of cervical pain radiating to the right arm. She had no history of other illnesses, surgeries, related traumas, or a family history of spinal diseases. No skin pigmentary abnormalities were observed. Neurological examination revealed mild weakness in the right arm and dysesthesia in the C5-C6 right dermatome. Electromyography (EMG) testing was performed and did not disclose any abnormality. Spinal cord Magnetic Resonance Imaging (MRI) demonstrated two right intracanal extradural and intra/extra-foraminal lesions at the cervical level: the first lesion measured 20x10mm and was located at the C6-C7 level with paravertebral extension and “dumbbell” shape; the other smaller one was located at C5-C6 level (**Figure 2**).

**Figure 3:**
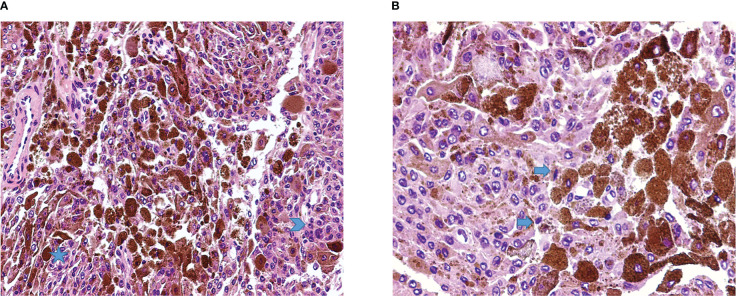
**(A)** Malignant melanotic nerve sheath tumor (MMNST) composed by pleomorphic cells, with spindle to polygonal shape, organized on fascicles (star) or sheets of roughly syncytial epithelioid cells (arrowhead), and numerous elements with heavy pigment deposits (H&E, magnification 20x). **(B)** Cellular details show the presence of occasional mitoses (arrows) and brown finely granular melanin pigment (H&E, magnification 40x).

The case was discussed by a multidisciplinary team which set the indication for surgical removal of the lesions. The patient gave written informed consent for the procedure. On the day of surgery, the patient was positioned supine, and a laminectomy was performed at the C5-C7 levels. Through a microscopic technique and an extended transcanalar approach, a GTR of intracanal, intra-foraminal, and paravertebral components of the two lesions was performed. Fat tissue was placed to reinforce the dural closure. The postoperative course was uneventful and characterized by strength recovery. On the other hand, dysesthesia slightly improved, but never recovered completely. The patient underwent a postoperative cervical MRI, which confirmed radical excision of both lesions (**Figure 2**). Surgical specimen underwent histopathological examination, which showed spindle-shaped epithelioid cells with eosinophilic cytoplasm carrying abundant melanin and scattered psammoma bodies. Neoplastic cells featured nuclear polymorphisms and evident nucleoli as well (**Figure 3**). Immunochemistry showed cellular positivity for “ HMB-45, S100, Synaptophysin, Melan-A and Collagen-IV “, thereby confirming a diagnosis of MNNST. MIB-1 proliferative index was 4-5%. To rule out CC, molecular screening on *PRKAR1A* gene was performed using Sanger sequencing, and no germline pathogenic variant in *PRKAR1A* was identified. Concerning the young age of the patient, research on the pathogenic variant in phacomatosis susceptibility genes (*NF1, NF2, SPRED1, SMARCB1, LZTR1)* and other 24 genes relevant in the pathogenesis of nervous system tumors (*TP53, EGFR, PDGFRA, PTEN, PIK3CA, PIK3R, RB1, NOTCH1, CDK4, CDKN2A, CDKN2B, IDH1, IDH2, FUBP1 CIC, TACC3, TERTp, ATRX, DAXX, FGFR3, ACVR1, H3F3A, KIAA1549 BRAF)*, was performed using a customized gene panel on lymphocyte DNA and tumor tissue. No pathogenic variants of those genes were detected in both tissues. 30 days after the surgical procedure, a whole-body Fluorodeoxyglucose**-**positron emission tomography/computed tomography (FDG-PET/CT) was performed, showing no residual focal pathology nor metastasis, and an echocardiogram revealed no cardiac myxoma. One year after surgery, the patient was in clinical and radiological remission without any signs of recurrence of the disease as confirmed by a recent one-year FDG-PET/CT.

## Discussion

SSP-MMNST is a rare, aggressive, and diagnostically challenging tumor of spinal nerves. We reviewed cases of SSP-MMNST by performing a literature search on PubMed and Embase databases. Their features are summarized in **Table 1**. It should be noted that some case reports did not include complete clinical, treatment, and genetic data of patients.

**Table 1:** Reported cases of sporadic spinal psammomatous melanotic schwannoma.

Authors	Year	Sex	Age	L	NR	Clinical	Met	T	R	O
Killeen et al. (18)	1988	F	26	S	S1	5-year lombalgia and leg pain	NA	GTR	NO	Resolution
Hollinger et al. (19)	1999	M	47	T	T12	3-year back and leg pain	NO	GTR	NO	Resolution
Vallat-Decouvelaere et al. (20)	1999	F	35	L	L4	3-year lombalgia	Bone, lymph nodes	GTR	6-year	72/DOD
Vallat-Devouvelaere et al. (20)	1999	M	27	L	L5	lombalgia	Lung, pleura	GTR	NO	72/DOD
Cummings et al. (21)	2000	M	51	S	S2	8-month lombalgia	NA	Declined Surgery	NA	NA
Kuchelmeister et al.(22)	2004	F	53	C	C6	2-year brachialgia with radicular radiation of pain into the right digits I-III	NO	GTR	NO	Resolution
Buhl et al.(23)	2006	M	28	L, S	L5-S1 + multiple small lesions	4-week lombalgia and sciatalgia	NO	GTR	2,5-year	30/AWD
Marton et al. (24)	2007	F	30	C	C3	6-month neck pain and spasms	NO	GTR	12-month	12/AWD
Azarpira et al.(25)	2009	M	37	L	L2	8-month lombalgia	NA	GTR	NA	NA
Arvanitis (14)	2010	M	36	L	L3	Back pain, weakness, weight loss	NA	STR x2	NA	NA
Izquierdo et al.(26)	2010	F	29	T	T8	Leg paresthesia, gait disturbance, muscle spasms	NO	GTR	NO	Resolution
Zhao et al. (27)	2011	M	46	C	C7	1-year neck pain, hand weakness	NO	GTR+RAD	NO	Resolution
Shields et al. (11)	2011	F	65	T	T7	Back pain	NO	STR+RAD	8-month	8/DOD
Shields et al. (11)	2011	M	33	L	L5	Lombalgia with radicular irradiation on the leg	Lung	STR+RAD	2-year	48/DOD
Mahesh et al. (28)	2014	M	67	T	T8-T12	2-week paraparesis, constipation, dysuria	NO	GTR	NO	Resolution
Bakan et al. (15)	2015	F	31	T	T4	Back pain	NO	GTR	NO	Resolution
Shabani et al. (29)	2015	M	54	C	C5	Incidental, on monitoring developed cervicobrachialgia	Lower spinal nerve root	GTR	3-month	7/DOD
Guzel et al. (17)	2016	M	36	L	L5	3-month lombalgia	NO	GTR	NO	Resolution
Khoo et al. (30)	2016	F	36	L	L5	4-year hip and leg pain	Brain and meninges	STR x2	10-month	NA
Mahmood et al.(31)	2016	M	17	T	T3	6-months pain and discomfort in the right upper chest	NO	GTR	NO	NA
Takatori et al.(32)	2020	M	39	L	L4	Lombalgia, numbness	Lungs, spinal cord, chest wall, stomach	STR+RAD	NO	22/DOD
Nagashima et al. (33)	2020	M	48	S	S2	6-month lombosciatalgia, dysuria	NO	GTR	NO	Resolution
Yeom et al. (34)	2022	F	58	T	T11-T12	Low back pain, paresthesia and cold sensation in both legs for years	NO	GTR	NA	NA

L, location; NR, nerve root; Met, metastasis; T, treatment; R, recurrence; O, outcome; DOD, Death of Disease; AWD, Alive with Disease; RAD,Radiation; NA, Not Applicable.

MMNST, formerly known as Melanotic Schwannoma or Schwannian melanotic tumor, was classified as a malignant lesion in the 2020 WHO classification of soft tissue tumors (4). The neoplasm rarely affects children and most commonly arises in adults, presenting as a sporadic lesion or as part of CC. The mean age of presentation is 38 years and no geographic, racial, ethnic, or gender preferences have been reported so far (35, 36). MMNST development in younger patients may be related to Carney syndrome (24). Psammomatous variants may be seen in patients with CC and arise a decade earlier than isolated sporadic cases, with a prevalence peak in the third decade of life (24, 37). In our analysis, the average disease age of onset for SSP-MMNSTs was 40±13 years (range 17 to 65 years), with slight male prevalence (1.56:1, M:F ratio). This is in contrast with previous studies in which no significant sex predilection was reported.

MMNST generally arises from spinal nerves and sympathetic ganglia as a single lesion (6, 9, 23). Nonetheless, literature reports scarce occurrences in other sites such as sympathetic chains, cranial nerve roots, peripheral nerves, cerebellum, orbit, choroids, soft tissue, alveolar nerves, palate, parotid gland, heart, oral cavity, oesophageal wall, pancreas, trachea, and bones (2, 10, 38–45). Spinal MMNST occurs in the lumbosacral region in 47.2% of cases, in the thoracic tract in 30.5%, and in the cervical region in 22.2%. The tumor may grow both in an intramedullary and extramedullary pattern. Furthermore, the lesion usually moves into the vertebral foramen or paravertebrally mainly affecting the posterior nerve roots (6, 7, 20, 46–48). Our review focused on SSP-MMNST and showed that the lumbar district is the most affected one, with no significant difference between the right and left sides.

The clinical presentation of spinal MMNST is related to its anatomical location. Approximately one-third of cases are asymptomatic (9). On the contrary, symptoms of spinal nerve and spinal cord involvement, which are observed in 35.5% of patients, include radicular and back pain, dysesthesia, progressive sensory and motor deficits, ataxia, and sphincter disorders (38). The peculiar combination of radicular and back pain leads to misdiagnosis, with discopathy being the most frequent one. Mechanical dysfunction of adjacent organs due to compression is reported in 13% of cases (49). Complications vary according to tumor location and may include CC sequelae such as heart failure and stroke (1). Our case was characterized by mild weakness in the right arm and dysesthesia in the C5-C6 right dermatome with progressive deterioration within a year.

Unlike common schwannomas, MMNST is prone to local recurrence and displays metastatic potential (6, 9, 39). Metastases primarily occur in the lung and pleura but may also involve mediastinum, diaphragm, pericardium, endocardium, bone, liver, and spleen (6, 9, 38). In their studies, Torres-Mora et al. and Alexiev et al. showed local recurrence in 15 to 35% of cases and a metastasis rate of 26% to 44% (1, 6).

Our review on SSP-MMNST demonstrated metastasis occurrence in 31.58% of cases, with male prevalence (66.6%) and with no statistically significant difference from the metastatic rate of spinal MMNST (p=0.463) (40). The main metastatic site was lung parenchyma, consistent with previous literature.

In 83% of cases of metastasis, the primitive tumor developed in the lumbar region with a slightly greater risk in males (RR=1.45, p=0.608). The mean age in patients with metastatic disease was lower than non-metastatic ones (37.33 ± 9 vs 42 ± 15 years), with no significant difference.

Comparing whole-body MMNST data (6) with ours on SSP-MMNST, we observed no significant difference in the metastasic rate (44% vs 31.58%, p= 0.563) nor in the recurrence rate (35% vs 36.84%, p = 0.8833). Furthermore, when considering only sporadic spinal MMNSTs (40) and comparing them with our SSP-MMNSTs, we did not find a significant difference in the rate of metastasis (32.7% vs 31.58%, p= 0.9273) nor recurrence (25% vs 36.8%, p= 0.2639). These results seem to support the hypothesis that this histological type has no greater local or distant aggressiveness than sporadic and spinal non-psammomatous cases and whole-body MMNST. We believe that the lack of significant difference between the spinal sporadic MMNST cases and our SSP-MMNST ones may arise from the small sample size available. Nevertheless, the recurrence rate of 36.8% in SSP-MMNST versus 25% in sporadic spinal MMNST appears to be an interesting finding that, if confirmed, could reveal a greater aggressiveness of the former.

Histologically, MMNST generally consists of a single ovoid lesion, and it is rarely multifocal. Adjacent bone erosion may be observed (6, 9, 23, 41). Although being a circumscribed tumor, MMNST does not feature any capsule, in contrast to the typical schwannoma. This characteristic may reflect the potential aggressiveness of the disease. Microscopically, spindle-shaped and plump epithelioid cells appear in intertwined fascicles or nests (1). Melanin accretions in the neoplastic cells and associated melanophages are usually identified. Cytoplasmic pigmentation is highly variable. The pigment is positive for silver Fontana-Masson melanin staining and negative for Prussian blue and periodic acid-Schiff (PAS) staining (50). Rare mitoses are discernible in most lesions. MMNSTs typically lack Verocay bodies, microcysts and thick-walled hyalinized blood vessels (42). The criteria for malignancy in MMNST are not yet fully established. Nonetheless, histological characteristics like large vesicular nuclei with macronucleoli, intense mitotic activity, and necrosis point towards aggressive behavior (6, 38). Immunohistochemical staining for S100, SOX10, HMB-45, Melan-A, p16 and vimentin yields positive results, whereas GFAP, EMA, and CK staining are mostly negative (6, 9, 20, 41–45, 49, 50). All MMNST cases display positive laminin and collagen IV linear and pericellular immunoreactions (50). Aside from our case report, we identified two other MMNST cases with a hemorrhagic presentation, one of which was a heart attack mimicker (27, 40). In our case, the histological features matched with data available in the literature (**Figure 3**).

MMNST pathogenesis is still poorly understood. Several theories about histogenesis of the tumor have been described. Schwann cells and melanocytes share both their neuroectodermal origin and migration routes. Given this common developmental lineage, Schwann cells may be capable of synthesizing melanin under specific circumstances (18, 51, 52). Further hypotheses point to a melanomatous transformation of neoplastic Schwann cells (53).

Genetically, MMNST features a complex karyotype with recurring 22q band monosomy, variable whole chromosomal gains, and recurrent losses usually affecting chromosomes 1 and 21 and chromosome arm 17p.45 (54). Approximately half of the psammomatous cases are associated with CC, an autosomal dominant genetic disease harboring a chromosome 17 mutation that affects the cAMP-dependent protein kinase type I regulatory subunit alpha (*PRKAR1A*) gene (9, 42). CC diagnosis requires at least two major clinical findings. However, a single finding is sufficient in cases of positivity for the inactivating *PRKAR1A* gene mutation (38). MMNST may also develop in neurofibromatosis type I (12, 48). Recent cytogenetic studies demonstrated trisomy 6p at ring chromosome 11 in MMNST, suggesting some similarities with malignant melanoma. However, MMNST lacks the prototypical BRAFV600E mutation (55).

MMNST diagnosis is based on clinical, histopathological, and instrumental findings. The histological diagnosis requires careful differential considerations between neurofibroma, pigmented dermatofibrosarcoma protuberans, melanocytoma, and malignant melanoma.

Our case matched previous literature, with both tumors being positive for HMB-45, S100, Synaptophysin, Melan-A, and Collagen-IV. MIB-1 proliferative index was 4-5%. Phacomatosis susceptibility and CNS tumorigenesis genes were also investigated with negative results. Their negativity allowed us to exclude melanoma, CC, and Neurofibromatosis.

Radiologically, SSP-MMNST is characterized by hyperintensity in T1-weighted sequences, hypointensity in T2-weighted sequences, and homogeneous post-contrast enhancement (**Table 2**). These features were confirmed in our case (**Figure 2**). By contrast, schwannomas often present hypointensity in T1 and hyperintensity in T2 (56–58). Melanin displays paramagnetic effects, leading to stable free radicals. Essentially, melanin protons have shorter T1 and T2 relaxation times. Nevertheless, melanin concentration and tumor density are not always consistent. On axial images, the tumor may appear dumbbell-shaped with varying relationships to the medulla and dura. According to the Asazuma et al. classification (59), dumbbell schwannomas are classified into 6 types with our case classified as type IIb (Extradural, inside the spinal canal, intraforaminal and paravertebral).

**Table 2:** Comparison of Spinal MMNST and SSP-MMNST.

	Spinal MMNST	SSP-MMNST
Epidemiology	35-55 years	40±13
Associated syndromes	Carney’s complex	Sporadic: PRKAR1A, NF2 and BRAFv606 negative. No evidence of skin lesions, myxomas nor endocrinological tumors.
Location	Thoracic (33.8%), lumbar (27.3%), cervical (26%), sacral (13%)	Lumbar (37.5%), thoracic (29.17%), cervical (16.67%), sacral (16.67%)
Sign and symptoms	Dysesthesia, pain	Dysesthesia, pain
Neuroradiology	T1 hyperintense, T2 hyperintense	T1 hyperintense, T2 hyperintense
Treatment	GTR if symptomatic, consider adjuvant chemotherapy or radiotherapy	GTR if symptomatic, consider adjuvant chemotherapy or radiotherapy
Recurrence	25%	36.8%
Metastasis	32.7%	31.5%

FDG PET/CT provides a unique contribution to: (a) the differential diagnosis of benign and malignant lesions, (b) the detection of covert metastases, (c) the monitoring of treatment response, and (d) the evaluation of MMNST prognosis (6, 9, 39, 60, 61). The patient in our case underwent two full-body FDG-PET-CT scans, the first in the immediate post-operative period and the second at one-year follow-up. In both cases, the scan was negative for focal uptake areas, ruling out disease recurrence and metastasis.

Surgery represents the cornerstone of treatment for MMNST, with GTR being the gold standard for all subtypes. During surgery, a residual tumor capsule may be left to prevent spinal cord injury if the tumor is closely adherent (62, 63).

In our review on SSP-MMNST, the most common approach was GTR in 73.91% of cases. Among these, only 23.53% developed disease recurrence, as opposed to 60% of patients who underwent STR. The total recurrence rate was 36.84%, and 57.14% of that percentage was associated with metastasis. This finding may suggest that recurrent SSP-MMNSTs tend to be more aggressive and, therefore more prone to spread systemically.

Given the limited number of cases, post-surgical management is controversial. No current guidelines for adjuvant treatment in MMNST are available (38, 50). Some studies show a beneficial role of adjuvant radiotherapy in post-operative tumor residuals, reducing the rate of relapse and metastasis during a 24-month follow-up (50). Fractionated radiotherapy is a suitable option for complex MMNST close to susceptible structures, such as the spinal cord, although no significant data on mortality reduction are yet available (38). Indeed, some studies have recommended 54 Gy fractionated radiation therapy applied to the spine (46). Spinal stereotactic radiosurgery may also be considered. Chemotherapy has instead demonstrated low response rates and no mortality benefit (64). In our analysis, radiotherapy was employed in only 18.18% of cases as an adjuvant treatment following STR. Given the metastatic potential of SSP-MMNST, these data seem to suggest that, whenever residual disease is left, adjuvant radiotherapy may be appropriate.

## Conclusion

Differently from common schwannomas, MMNST represents a rare disease with known recurrence and metastatization propensity. As reported in our review, SSP-MMNST has a greater recurrence rate when compared to other forms of spinal MMNST, raising questions about its greater aggressiveness. We also found that residual disease is related to a higher risk of systemic spreading. This metastatic potential, usually associated with primary lumbar localization, is characterized by a slight male prevalence. Indeed, whenever GTR is unachievable, considering the higher recurrence rate, adjuvant radiation therapy should be taken into consideration.

## Data availability statement

The original contributions presented in the study are included in the article/supplementary material. Further inquiries can be directed to the corresponding author.

## Ethics statement

Ethical review and approval was not required for the study on human participants in accordance with the local legislation and institutional requirements. The patients/participants provided their written informed consent to participate in this study. Written informed consent was obtained from the individual(s) for the publication of any potentially identifiable images or data included in this article. Patient signed informed consent regarding publishing his data and photographs.

## Author contributions

GB, EM, and MB performed the clinical assessment. GB, AG, EM, ER, PA, and GI critically reviewed the literature and drafted the manuscript. All authors were responsible for important intellectual content. All authors contributed to the article and approved the submitted version.
